# Non-target time trend screening: a data reduction strategy for detecting emerging contaminants in biological samples

**DOI:** 10.1007/s00216-016-9563-3

**Published:** 2016-04-27

**Authors:** Merle M. Plassmann, Erik Tengstrand, K. Magnus Åberg, Jonathan P. Benskin

**Affiliations:** Department of Environmental Science and Analytical Chemistry (ACES), Stockholm University, Svante Arrhenius Väg 8, 10691 Stockholm, Sweden

**Keywords:** Biological samples, Non-target screening, Data processing, Time trend filtering, Emerging environmental pollutants

## Abstract

Non-targeted mass spectrometry-based approaches for detecting novel xenobiotics in biological samples are hampered by the occurrence of naturally fluctuating endogenous substances, which are difficult to distinguish from environmental contaminants. Here, we investigate a data reduction strategy for datasets derived from a biological time series. The objective is to flag reoccurring peaks in the time series based on increasing peak intensities, thereby reducing peak lists to only those which may be associated with emerging bioaccumulative contaminants. As a result, compounds with increasing concentrations are flagged while compounds displaying random, decreasing, or steady-state time trends are removed. As an initial proof of concept, we created artificial time trends by fortifying human whole blood samples with isotopically labelled standards. Different scenarios were investigated: eight model compounds had a continuously increasing trend in the last two to nine time points, and four model compounds had a trend that reached steady state after an initial increase. Each time series was investigated at three fortification levels and one unfortified series. Following extraction, analysis by ultra performance liquid chromatography high-resolution mass spectrometry, and data processing, a total of 21,700 aligned peaks were obtained. Peaks displaying an increasing trend were filtered from randomly fluctuating peaks using time trend ratios and Spearman’s rank correlation coefficients. The first approach was successful in flagging model compounds spiked at only two to three time points, while the latter approach resulted in all model compounds ranking in the top 11 % of the peak lists. Compared to initial peak lists, a combination of both approaches reduced the size of datasets by 80–85 %. Overall, non-target time trend screening represents a promising data reduction strategy for identifying emerging bioaccumulative contaminants in biological samples.

**Electronic supplementary material:**

The online version of this article (doi:10.1007/s00216-016-9563-3) contains supplementary material, which is available to authorized users.

**Graphical abstract:**
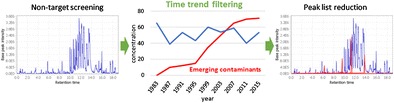
Using time trends to filter out emerging contaminants from large peak lists

## Introduction

Most approaches for screening environmental contaminants target individual chemicals or chemical classes using highly specific analytical methods. Despite their utility for low-level detection and quantification, these methods often overlook novel contaminants or transformation products which may pose a risk to humans and wildlife. Recent advances in mass spectrometry and chemometrics have addressed this limitation through development of non-targeted screening approaches, in which samples are analyzed without a priori knowledge of the contaminants of interest [1]. Non-targeted methods involve broad sample extraction procedures combined with gas or liquid chromatography high-resolution mass spectrometry (GC- or LC-HRMS, respectively), advanced data processing tools, and identification by comparison with mass spectral libraries and structure elucidation.

Among the principal challenges of a purely non-targeted screening approach is the number of peaks present in datasets, which can be on the order of several thousands per sample [2, 3]. Therefore, processing of non-targeted datasets is time intensive, making it advantageous to reduce the number of relevant peaks prior to attempting compound identification. Current strategies for data reduction include flagging peaks with chlorine and bromine isotopes or retention time homologues, and statistical comparisons, in which peaks absent in controls are selected for further investigation. While demonstrating great potential for screening water [2, 4–7] and sediment samples [8], applications to biological matrices are less common [9–11]. This is likely due to the ubiquity of endogenous substances (i.e., metabolites), which are not easily differentiated from xenobiotics [3] and are present at substantially higher concentrations in blood samples compared to xenobiotics [12].

An alternative data filtering strategy—specific to chronological datasets—involves flagging important chromatographic peaks based on their systematic (i.e., non-random) fluctuation over time. This approach was applied for narrowing down transformation products and metabolites in river sediments [13], and a similar feature is offered through the software package SIEVE (Thermo Fisher Scientific Inc., USA), which can allow the user to assess data based on intensity or trend ratios. Despite showing considerable potential for identifying important features in a chronological dataset, these approaches rely on visual assessment of trends, which is not practical for filtering the thousands of peaks obtained from non-targeted analysis of biological samples. A more automated, statistically based approach was reported by Peters et al. [14] in which curve fitting and autocorrelation algorithms were applied to detect non-random variation in metabolite levels, resulting in a >98 % data reduction. While showing great promise for metabolomics (where both increasing and decreasing trends are important), this approach may not be appropriate for emerging bioaccumulative contaminants, which are expected to only display increasing time trends.

In the present work, we investigated an automated, statistically based data reduction strategy for identifying emerging bioaccumulative contaminants using increasing peak intensities over time. Decreasing trends were not included since they are less relevant to emerging contaminants, but could be investigated simply by reversing the sample order. As an initial proof of principle, human whole blood samples were fortified with isotopically labelled xenobiotics to create different time trends. Following extraction, analysis by ultra performance liquid chromatography (UPLC)-HRMS, and peak alignment, two statistical approaches were employed. The extent of data reduction was assessed, as well as the efficacy of each method for filtering model compounds.

## Materials and methods

### Standards and reagents

Standards of caffeine-d_9_, sulfamethoxazole-d_4_, bezafibrate-d_5_, diflufenican-d_3_, metoprolol-d_7_, sotalol-d_6_, propranolol-d_7_, fluoxetine-d_5_, diatrizoic acid-d_6_, glimepiride-d_5_, ranitidine-d_6_, and acetaminophen-d_4_ were obtained from Toronto Research Chemicals (Toronto, Canada). Labelled standards were chosen due to availability and their suitability towards the analytical method. Human whole blood samples from nine anonymous individuals were obtained from Karolinska Institutet (Stockholm, Sweden) in accordance with ethical guidelines set by the Swedish ethics committee.

### Sample preparation

**Fig. 1:**
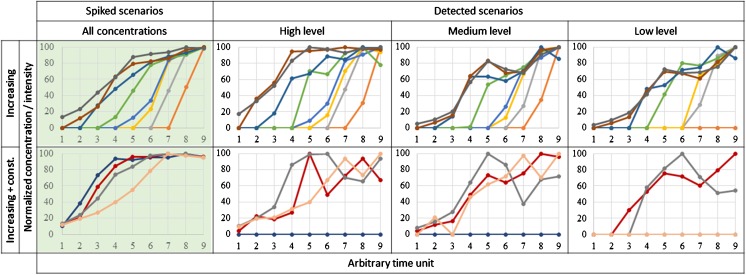
Comparison between spiked time trend scenarios and the scenarios detected after LC-MS analysis and data processing at three different spike levels. Each *color* represents one spiked compound; for names, see Table 1. The spiked concentrations and the detected intensities are normalized to 100 % of the maximum value

Spiking scenarios represented either increasing trends starting at different time points using caffeine-d_9_, sulfamethoxazole-d_4_, bezafibrate-d_5_, diflufenican-d_3_, metoprolol-d_7_, sotalol-d_6_, propranolol-d_7_, and fluoxetine-d_5_ or trends which increased initially and then plateaued using diatrizoic acid-d_6_, glimepiride-d_5_, ranitidine-d_6_, and acetaminophen-d_4_. Concentrations increased by a factor of two to ten over the course of the time trend. In order to introduce variability into the dataset, each of the nine individual blood samples were used as a different time point (arbitrary time trend increments of 1 to 9 [unitless]). The number of samples in the time series was selected in order to have a sufficient number of time points to generate a robust time trend, but so as not to generate an excessively large peak list. Of the nine samples, three artificial time trends were prepared by fortifying blood samples (1 mL each) with labelled standards at high (10–100 ng/mL), medium (2–20 ng/mL), and low (0.2–2 ng/mL) concentration ranges. An additional series was prepared without fortification with labelled standards (blank series). Spiked trends normalized to 100 % of the highest concentration are presented in Fig. 1, and exact fortification levels can be found in Table S1 in the Electronic Supplementary Material (ESM).

The blood samples were extracted according to a previously tested method [15] which involved liquid-liquid extraction with 2 mL of acetonitrile (ACN), 0.4 g of MgSO_4_, and 0.1 g of NaCl. Three stainless steel beads (3.2 mm diameter) were added, and the mixture was placed into a bead blender (1600 MiniG®, SPEX SamplePrep, USA) for 30 s at 1500 rpm, followed by centrifugation at 2500 rpm. An aliquot of the supernatant (1.6 mL) was concentrated to dryness by N_2_ and reconstituted in 80 μL of ACN/H_2_O (1/1).

### Instrumental analysis

Analysis was performed using an Acquity UPLC coupled to a Xevo G2-S quadrupole time-of-flight (QTOF) mass spectrometer (Waters) via an electrospray ionization source operated in positive mode. The instrumental analysis method was adapted from methods previously used in a collaborative trial on non-target screening of water [2]. Five microliters of extract was injected onto an Acquity UPLC HSS C18 SB column (2.1 × 100 mm, 1.8 μm) maintained at room temperature. Separation was achieved using a 19-min gradient from 95 % H_2_O (5 mM ammonium formate, 0.01 % formic acid) to 99 % ACN (0.01 % formic acid) with a flow of 0.5 mL/min (plus a 2-min equilibration time). The mass spectrometer was operated in full scan (100–1000 Da) with a scan time of 0.25 s and a collision energy of 4 eV.

### Data processing

Data processing was conducted using the software TracMass2 [16], running under MATLAB (MathWorks®, USA). Parameters used for peak detection and alignment are listed in Table S2 (ESM). Peak lists containing aligned peaks were created for each spike level and one containing all 36 samples. To reduce the number of false positives, peaks detected in a single sample were not included. Statistical analysis was conducted in MATLAB and Microsoft Excel.

Two statistical approaches were tested, one based on comparison of average intensities in two sample sets and one testing the increasing trend by application of Spearman’s rank correlation coefficient. For each peak, the following calculations were performed: First, the average detected intensities at time points 7–9 were divided by the average detected intensities at time points 1–6 (+1 to avoid dividing by 0). We defined this value as the “time trend ratio (TTR).” A high TTR—representing a possible emerging bioaccumulative contaminant—is produced by peaks with low intensities in early samples and high intensities in later samples of the time trend. Second, Spearman’s rank correlation coefficient was calculated for all peaks with detections in at least three samples in the time trend. This results in a value close to 1 for peaks with a monotonically increasing time trend. Peaks in the full peak lists were subsequently ranked according to calculated TTR and Spearman’s rank correlation coefficients (*ρ*).

## Results and discussion

### Detection

The nine blood samples were extracted and analyzed four times each, for a total of 36 analyses. The number of total aligned peaks detected in each artificial time trend series (detection in ≥2 of 9 samples) was 11,800, 11,400, 12,600, and 12,200 for the high, medium, low, and blank levels, respectively. When aligning all time trend series in one list, a total of 21,700 aligned peaks (detection in ≥2 of 36 samples) were obtained. The consistency in number of peaks arises from using the same blood samples for each time trend series. Following analysis by TracMass2, 11 of 12 spiked compounds (all except diatricoic acid-d_6_) were detected at the high and medium spike levels, and 8 were detected at the low spike level (not detected: diatricoic acid-d_6_, acetaminophen-d_4_, caffeine-d_9_, and diflufenican-d_3_). Spiked compounds were not detected in the blank series. The spiked and measured time trend scenarios are plotted in Fig. 1, showing reasonable consistency even at the low spike level.

To assess the distribution of replicate and biological variation in the dataset, Bayesian ANOVAs [17] were performed. The relative standard deviation (RSD) of the four replicates (three spike levels and one blank) was compared to the RSD of the nine individual samples (biological RSD; see Fig. S2, ESM). The replicate RSD was on average 21 %, with 90 % of peaks displaying RSDs of 6–47 %. In contrast, the average biological RSD was 24 % but showed a much broader range (1–77 %), indicating that replicate variation in the data is about the same as the variation between samples from different persons. Therefore, for the analysis of real time trend samples, several replicate analyses should be conducted to reduce uncertainty in the detected intensities. The use of quality control samples and internal standards has been described as another means of reducing analytical variability [18]. Additionally, the repeated analysis of pooled samples could reduce the variability of both the endogenous and exogenous compounds present at each time point. On the other hand, pooling samples in a longitudinal study should be conducted with caution as this can result in a loss of information.

### Ranking

Ranking peaks for the entire spiked time trend series according to the TTR or *ρ* values resulted in a high rank for each of the spiked compounds using at least one of the two methods. The calculated TTR and *ρ* values and the resulting ranks for the spiked compounds at the high spike level can be found in Table 1, while the data for the other two spike levels are listed in Table S3 (ESM).

**Table 1:**
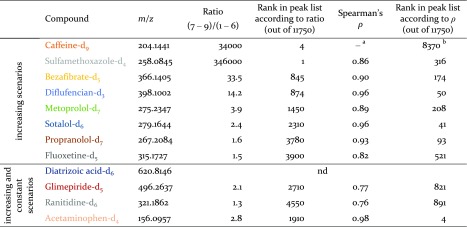
Time trend ratios (TTR), Spearman’s *ρ*, and resulting ranks of spiked compounds in the peak list of the high-spike-level artificial time trend. The colored names represent the scenarios in the same colors of Fig. 1

*nd* not detected

^a^ *ρ* values were only calculated for peaks with more than two detections in the time trend; thus, no value resulted here

^b^Peaks without a *ρ* value were sorted after the others according to their *m*/*z* values

The TTR calculated by comparison of average intensities was particularly effective at ranking spiked compounds only present at two to three of the latest time points in the time trend high on the list, i.e., caffeine-d_9_ and sulfamethoxazole-d_4_. These two compounds showed substantially higher ratios than other spiked compounds present at more than three time points. This calculation is thus an efficient method to filter out substances appearing in recent years (i.e., emerging bioaccumulative contaminants), which may thus far have not been discovered. The TTR comparing the latest three with the first six time points ranks those compounds only present at one to three of the latest time points high on the list. When changing the TTR to comparing the latest four time points with the first five instead, the ranks of the compounds present at the latest four time points were increased; however, the rank of caffeine-d_9_ (present at the two latest time points only) was decreased. This thus includes more compounds appearing at a wider time span. Which TTR to use for future applications is thus dependent on the number of time points and the span of years that are covered.

Using Spearman’s rank correlation coefficient, ten spiked compounds received *ρ* values of ≥0.76 at the high spike level (≥0.76 and ≥0.72 at the medium and low spike levels, respectively); no value was calculated for caffeine-d_9_ as it was only detected in two samples. The magnitude of the concentration increase over the time course did not affect the *ρ* value since it was calculated based on ranks. Thus, *ρ* was solely affected by how well the increase showed a monotonic trend, as can be seen by comparing glimepiride-d_5_ and ranitidine-d_6_ with acetaminophen-d_4_. Glimepiride-d_5_ and ranitidine-d_6_ displayed random variation over the last five time points resulting in *ρ* values of 0.77 and 0.76, respectively, while acetaminophen-d_4_ displayed only one time point breaking a monotonically increasing trend, resulting in a *ρ* value of 0.98. When ranking according to *ρ* values, all ten compounds were in the top 8 % of the entire peak list at the high spike level (and in the top 6 and 11 % at the medium and low levels, respectively). Thus, this rank test appears to be a good test to filter out compounds when a general increasing trend is present at three or more time points.

### Peak list reduction

The two tested approaches filter out two different types of trends in the data: those associated with compounds which are predominantly present at some of the latest time points (using the TTR) or compounds with a general increasing trend (*ρ*). Thus, an assessment including both tests was conducted, which included all spiked compounds. The extent of data reduction based on the rankings using both TTR and *ρ* was assessed. All peaks with either a TTR of ≥10 or a *ρ* ≥ 0.7 were combined in a separate peak list and duplicates were removed. This resulted in combined peak lists of 1800, 1700, and 2600 peaks for the high, medium, and low spike levels, respectively. Compared to the full peak lists, this represented a data reduction of 85, 85, and 80 %, for high, medium, and low spike levels, respectively.

Clearly, in a scenario involving real (i.e., unfortified) time trend samples, greater variability in the dataset may be expected. However, this variability can be reduced through inclusion of multiple samples per time point or a pooled sample. Even with a larger margin of safety, we expect that the number of peaks in a peak list could be reduced substantially using non-targeted time trend screening. Future work will apply this approach to real time trend samples, where pollutants with known increasing time trends (e.g., perfluoroalkyl acids [19]) can be used to assess the TTR and *ρ* values for peak list cutoff.

Despite the peak list reduction, the number of peaks left after using the non-target time trend approach is still too large to be identified. Thus, on top of the approach tested here, peak lists need to be further reduced by assessing isotopic ratios, adducts, and in-source fragmentation [7], along with checking peaks against known metabolite databases (e.g., the human metabolome database [20]) to exclude endogenous compounds from the peak lists [3]. These approaches, combined with non-targeted time trend screening, have the potential to significantly reduce non-targeted datasets, allowing greater resources to be placed on identification using suspect lists, isotopic or homologue pattern, mass defects, fragmentation spectra, and finally the comparison with reference standards.

## Electronic supplementary material

Below is the link to the electronic supplementary material.

ESM 1 (PDF 443 kb)
