# Supplementary material for: Non-target time trend screening: a data reduction strategy for detecting emerging contaminants in biological samples
Source: Anal Bioanal Chem. 2016 Apr 27;408:4203–8. doi: 10.1007/s00216-016-9563-3 (PMC4875932; doi:10.1007/s00216-016-9563-3)
Supplement: Supplementary file 1 — (PDF 443 kb) [file 216_2016_9563_MOESM1_ESM.pdf]

Analytical and Bioanalytical Chemistry

Electronic Supplementary Material

Non-target time trend screening: A data reduction strategy for detecting emerging contaminants in biological samples

Merle M. Plassmann, Erik Tengstrand, K. Magnus Åberg, Jonathan Benskin

Table S1 Concentrations [ng/mL] spiked into the artificial time trend series for each compound. Concentrations given for the medium spike level. For the high spike level concentrations were a factor 5 higher, while for the low spike level the concentrations were a factor 10 lower

		arbitrary time point number								
compound	m/z	1	2	3	4	5	6	7	8	9
increasing scenarios										
Caffeine-d_9	204.1441	-	-	-	-	-	-	-	9.8	19.4
Sulfamethoxazole-d_4	258.0845	-	-	-	-	-	-	9.2	18.8	20
Bezafibrate-d_5	366.1405	-	-	-	-	-	4.8	16.8	19.6	20.4
Diflufenican-d_3	398.1002	-	-	-	-	2.4	6.6	16.4	18.2	19.6
Metoprolol-d_7	275.2347	-	-	-	2.8	9.4	16	17.8	18.6	20.6
Sotalol-d_6	279.1644	-	-	5.6	9.8	13.2	16.4	17.8	18.8	20.2
Propranolol-d_7	267.2084	-	2.4	5.2	12.6	15.8	16.4	17.2	19.4	20
Fluoxetine-d_5	315.1727	2.6	4.6	8.6	12.4	17.3	18	18.5	19.7	19.4
increasing and then staying constant scenarios										
Diatrizoic acid-d_6	620.8146	2.2	8	15.2	19.4	19.2	19.8	19.8	20.8	20
Glimepiride-d_5	496.2637	2.4	4.4	12.2	17.6	20	20	20.8	20.6	20
Ranitidine-d_6	321.1862	2.8	5	9.2	15.4	17.4	20.2	20.8	20.8	19.8
Acetaminophen-d_4	156.0957	2.4	4	5.6	8.2	11.4	16.2	20.6	20	19.6

Table S2 TracMass2 parameters used during data processing

**Tracker parameters**	
minLength	9
minIntensity	1000
mzTolerance	0.01
mzAnchor	400
mzTransformation	Sqrt
rawData_threshold	0
mzRange	[0 inf]
timeRange	[0 inf]
**Peak detection parameters**	
zafSigma	1
Zaf2Sigma	3
gaussSigma	0.4
nSignaltoNoise	10
stdFiltWidth	10
**Cluster 1 parameters**	
deltaTime	8
deltaMass	0.01
Warping parameters	
numPSplines	50
**Cluster 2 parameters**	
deltaTime	8
deltaMass	0.01

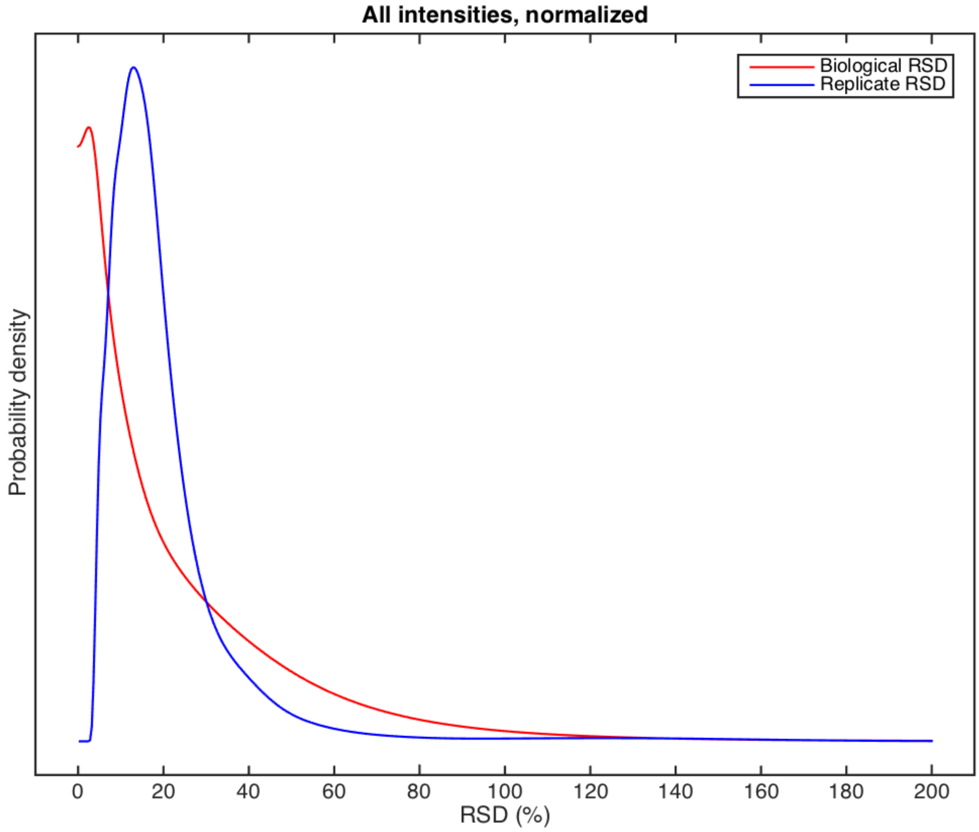

Fig. S1 Relative standard deviations (RSD) of detected intensities for 1497 peaks occurring in 35 out of 36 samples* calculated by Bayesian ANOVA for the four replicate samples in blue (replicate RSD) compared to the 9 individual samples in red (biological RSD). Intensities have been normalized by probabilistic quotient normalization [1], taking into account the decreasing intensities along the injection sequence

* One sample was an outlier and therefore removed from the analysis.

Table S3 Time trend scenarios (TTR), Spearman's ρ and resulting ranks of spiked compounds in the full peak lists of the medium and low level artificial time trends (total number of peaks and thus ranks for medium level: 11377 and for low level: 12624). The colored names represent the scenarios in the same colors of Figure 1 (nd = not detected)

Compound	m/z	TTR (7-9)/(1-6)		Rank in peak list according to TTR		Spearman's ρ		Rank in peak list according to ρ	
		medium	low	medium	low	medium	low	medium	low
Caffeine-d_9	204.1441	2541	nd	39	nd	-[a]	nd	8288[b]	nd
Sulfamethoxazole-d_4	258.0845	27070	2826	1	84	0.86	0.86	167	341
Bezafibrate-d_5	366.1405	41	1993	836	145	0.92	0.86	46	344
Diflufencian-d_3	398.1002	19.7	nd	845	nd	0.92	nd	54	nd
Metoprolol-d_7	275.2347	4.5	4.4	1254	1800	0.98	0.94	3	60
Sotalol-d_6	279.1644	2.6	3.0	2009	2271	0.93	0.97	40	12
Propanolol-d_7	267.2084	2.2	2.4	2417	2803	0.95	0.93	15	63
Fluoxetine-d_5	315.1727	2.1	2.3	2599	2980	0.93	0.95	28	33
Diatrizoic acid-d_6	620.8146	nd							
Glimepiride-d_5	496.2637	2.5	2.1	2085	3263	0.97	0.93	6	98
Ranitidine-d_6	321.1862	1.2	1.5	5075	4569	0.76	0.72	683	1438
Acetaminophen-d_4	156.0957	2.7	nd	1923	nd	0.93	nd	25	nd

[a] ρ values were only calculated for peaks with more than 2 detections in the time trend, thus no value resulted here;
[b] peaks without a ρ value were sorted after the others according to their m/z values

REFERENCES

1. Dieterle F, Ross A, Schlotterbeck G, Senn H Probabilistic Quotient Normalization as Robust Method to Account for Dilution of Complex Biological Mixtures. Application in 1H NMR Metabonomics. Anal Chem 2006; 78: 4281-4290.
